# Fast Regulation of Vertical Squat Jump during Push-Off in Skilled Jumpers

**DOI:** 10.3389/fphys.2016.00289

**Published:** 2016-07-19

**Authors:** Patrick Fargier, Raphael Massarelli, Tahar Rabahi, Angelo Gemignani, Emile Fargier

**Affiliations:** ^1^Inter-University Laboratory on Human Movement Biology (EA 7424), Centre for Interdisciplinary Research in Sport (FED 4272), University of Lyon, University Claude Bernard Lyon 1Villeurbanne, France; ^2^Laboratoire de Conception, Optimisation et Modélisation des Systèmes équipe émotion-action (EA 7306), Université de LorraineIle du Saulcy, Metz, France; ^3^Dipartimento di Patologia Chirurgica, Medica, Molecolare et dell'Area Critica, Università degli StudiPisa, Italy

**Keywords:** vertical squat jump, intersegment coordination, power, motor control, explosive movement

## Abstract

The height of a maximum Vertical Squat Jump (VSJ) reflects the useful power produced by a jumper during the push-off phase. In turn this partly depends on the coordination of the jumper's segmental rotations at each instant. The physical system constituted by the jumper has been shown to be very sensitive to perturbations and furthermore the movement is realized in a very short time (ca. 300 ms), compared to the timing of known feedback loops. However, the dynamics of the segmental coordination and its efficiency in relation to energetics at each instant of the push-off phase still remained to be clarified. Their study was the main purpose of the present research. Eight young adult volunteers (males) performed maximal VSJ. They were skilled in jumping according to their sport activities (track and field or volleyball). A video analysis on the kinematics of the jump determined the influence of the jumpers' segments rotation on the vertical velocity and acceleration of the body mass center (MC). The efficiency in the production of useful power at the jumpers' MC level, by the rotation of the segments, was measured in consequence. The results showed a great variability in the segmental movements of the eight jumpers, but homogeneity in the overall evolution of these movements with three consecutive types of coordination in the second part of the push-off (lasting roughly 0.16 s). Further analyses gave insights on the regulation of the push-off, suggesting that very fast regulation(s) of the VSJ may be supported by: (a) the adaptation of the motor cerebral programming to the jumper's physical characteristics; (b) the control of the initial posture; and (c) the jumper's perception of the position of his MC relative to the ground reaction force, during push-off, to reduce energetic losses.

## Introduction

Physical activities under extreme conditions or extreme sport disciplines have increasingly interested the world of sport in recent decades. It is easily understandable that climbing Mount Everest without additional oxygen supply (e.g., West, 1983), deep diving in apnea conditions (e.g., Muth et al., 2005) or performing in ironmen's triathlon competitions (e.g., Knechtle et al., 2010) bring athletes to their physiological and psychological limits and often beyond. In several cases and not only in sport but also in everyday life movements must be chosen and executed within a very short time under pressure and sometimes in a dangerous environment (as it may happen during automobile driving in intense traffic). The performance of physical activities possibly dangerous for body integrity strongly depends on a very accurate motor coordination, as for example in cliff diving, (e.g., Butterfield and Boyd, 2012). Sport in extreme conditions may thus lead to the study of individual psychological, physiological and biomechanical adaptations to specific constraints.

Under extreme conditions it is particularly necessary to develop the best possible coordination of bodily segments often to safely realize a good performance; possibly the best one. From this standpoint jumps have been frequently used to evaluate human physical capacities (notably from Sargent, 1921). In particular the performance of a Vertical Squat Jump (VSJ) is strictly bound to some qualities of the musculo-skeletal system that the jumper must optimally exploit to reach a maximum height of flight (Bobbert and van Soest, 2001). The height (h) of a jump is thus not only influenced by the power-generating capability of the muscles involved in the movement but also, and first of all, by the coordination of the jumper's segments during the push-off phase (e.g., Bobbert and van Soest, 2001) and by the segmental pattern of movement that is established in each individual.

In this context the pattern of movement of a given jump may be defined as the interaction of the muscular forces developed in response to the neural stimulation of the muscles with the mechanical constraints of the task (e.g., Bobbert and van Ingen Schenau, 1988). Jumpers who are experienced in VSJ develop a single pattern of movement, i.e., the way by which the rotation movements of the jumper's segments establish the vertical acceleration of the body mass center (MC). During the execution of a VSJ it has been observed that, in spite of time differences from an individual to another, the rotation of the segments implies the opening of the segmental and articular angles in a proximo-distal sequence (i.e., following the sequence trunk, thigh, leg, foot; e.g., Bobbert and van Soest, 2001). Such pattern has been considered to be the optimal sequence required to achieve a maximal jump height (Bobbert and van Soest, 2001) and it has also been found in other explosive movements, for example in the case of overarm throws (Atwater, 1979) or of counter-movement jumps (Bobbert and van Ingen Schenau, 1988). However, other patterns of movement may also be observed in VSJs, for example in elderly men where a simultaneous pattern of movement, and not a sequential proximo-distal pattern, has been reported (Haguenauer et al., 2005).

These observations regarding the establishment of a pattern of movement raise the problem of the coordination of the segmental movements and thus of the control of these movements during a push-off. An important issue, which has not yet been solved (Pinter et al., 2012). Time is a major point in the study of the motor control of a VSJ, as this is realized within ca. 300 ms. Such a time constraint places the jumper in a borderline condition to control his movements because (1) the physical system that constitutes the jumper is very sensitive to perturbation, and (2) the time of movement limits the possibilities to benefit from the known neural feedback loops (e.g., van Soest and Bobbert, 1993).

Even in the case of skilled jumpers it is probable that the pattern of the segmental and articular angles opening is not absolutely predetermined because of the intervention of different factors possibly fluctuating with time, such as muscular qualities or an exact initial posture, or the equilibrium, etc., Even the number of degrees of freedom of the brain sensorimotor system is likely to add some intra and inter-individual variability (Newell and Corcos, 1993). However, such potential variability does not interfere with the emergence of a consistent motor pattern, hence suggesting the existence of a robust process of regulation of the segment movements.

This process should be suitable for any jumper and should be revealed by the examination of the evolution of the segment coordination during the 300 ms push-off phase. This dynamics of coordination *per se*, leading to a proximo-distal pattern, remained however to be clarified and the aim of the present study was to determine such dynamics in the case of skilled jumpers. The examination of the segmental movements and of their interrelation was made in terms of kinematics but also in relation with the corresponding energetic outputs (i.e., jump efficiency) as a VSJ requires the best utilization of the produced muscular energy to reach the maximum height. This leads to the assumption that a possible regulation of the coordination should be function of this requirement.

## Materials and methods

**Figure 1:**
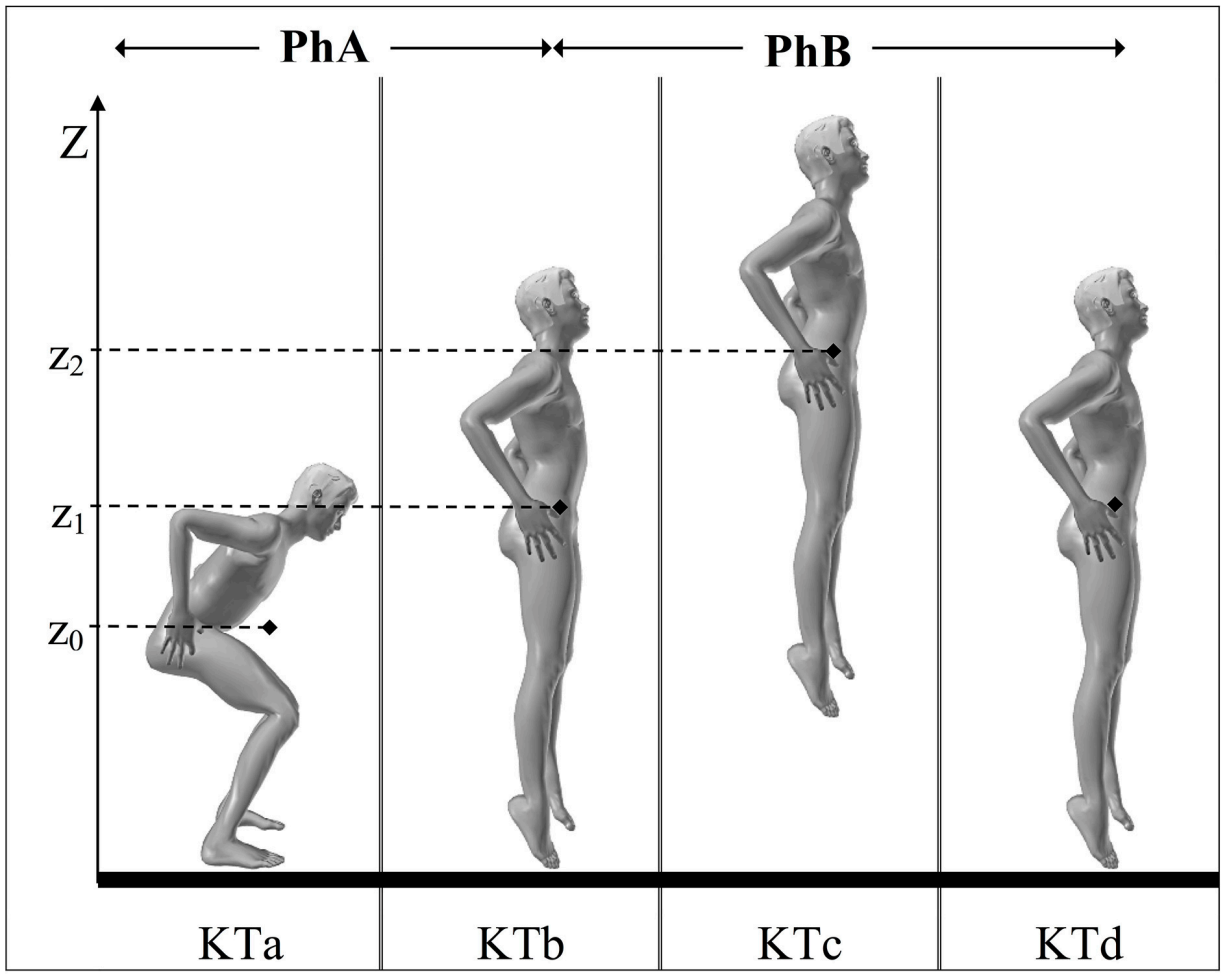
**Key times (KTa-d), phases (PhA-B), and performance (h) of a VSJ**. KTa: initial position (the jumper stands still, heels on the ground, knee joint angle of ~90°, hands on hips); KTb: take-off (end of contact between the support and the jumper); KTc: peak of the flight phase (the mass center of the jumper, MC, reaches its maximal height of flight); KTd: landing (moment at which the feet touch again the ground); PhA: push-off phase (realized without initial countermovement, it starts at t0 when the segments of the jumper begin to rotate and ends at take-off); PhB: flight phase (it starts at take-off and ends at the instant of landing). The height of the jump (h) is given by the difference between the vertical coordinate of MC (♦) at take-off (z1) and at the peak of the flight phase (z2): *h* = z2 − z1. During the push-off the coordinate of MC evolve from z0 (initial position) to z1 (instant of take-off) and then to z2 (apex of the jump).

The study was approved by the Institutional Review Board of Claude Bernard University Lyon 1 and the participants to the experimental protocol gave their informed consent. Eight male subjects (22.75 ± 3.3 years of age, 180 ± 0.06 cm height, and 71.87 ± 6.44 kg weight) participated to the study. The subjects were students of the Faculty of Sport Sciences (STAPS) skilled in either track and field jumps or in volleyball (French regional level) and had thus a consequent experience in jumps even if not familiar with VSJ. During the experiments the subject were asked to produce maximal VSJs (Figure 1), while respecting the following constraints: (1) a stationary semi-squatted initial position, (2) hands kept on the hips during the jump, (3) starting the push-off phase without any initial displacement of the jumper's MC toward the ground, consequently without any countermovement. Each VSJ was video-recorded and analyzed.

### Model of the jump

**Figure 2:**
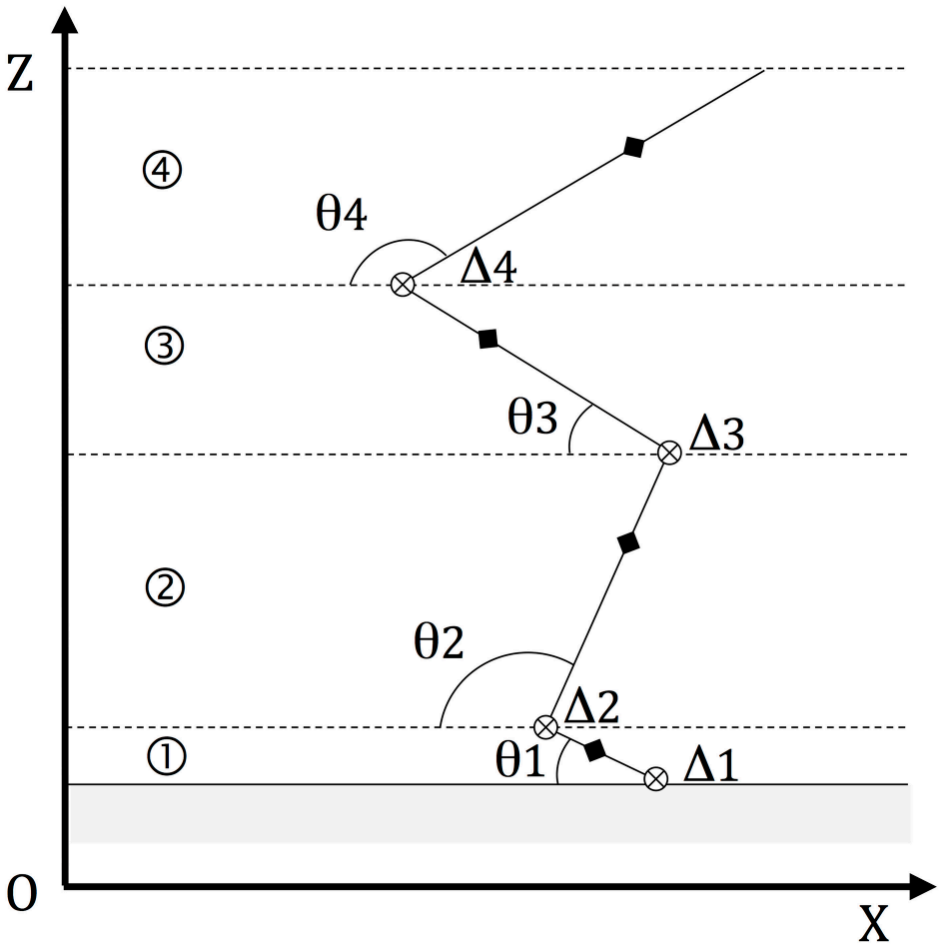
**Modeling the jump**. The model consists of four segments: ①, ②, ③, and ④ (respectively feet, legs, thighs, and head-arms-trunk). Other constituent elements are: the rotation axes orthogonal to (OX, OZ) (⊗), the segmental angles θi and the segmental mass centers MCi (♦).

The VSJ was modeled (Figure 2) as a planar, rigid body system represented by four segments (feet, lower legs, upper legs, and head-arms-trunk or HAT) linked by frictionless, hinge joints (e.g., Bobbert and van Soest, 2001; Babič and Lenarčič, 2007). The mass center of each segment (MCi) was found on the same vertical plane defined by a system of orthogonal axes (OX, OZ) related to the ground; the jumper's MC laid also on the same plane (Figure 2).

### Data acquisition

Before each experiment subjects were asked to execute few warm-up exercises and some VSJs to obtain a correct execution of the movement. They were subsequently asked to perform a maximum VSJs in 3 trials.

Five optical markers were placed, on the gleno-humeral joint (shoulder), the great trochanter (hip), the lateral condyle of the femur (knee), the lateral malleolus (ankle), and the fifth metatarsal (toe) (e.g., Aragón-Vargas and Gross, 1997). Subjects were filmed on the right sagittal plane with a camcorder (JVC©, GR-DVL 9200) at a distance of 7 m. The angle between the optical axis of the camcorder and the plane of movement was 90°. The video sequences have been deframed with the software Adobe Première© to obtain an image frequency of 50 Hz.

Video images were digitized using self-developed software. The coordinates for the markers were obtained from digitization of the central point of the landmarks and the coordinates, as function of time, were smoothed by using a moving average of order 1. The anthropometric values of the segments required for the analysis of the data were obtained from Winter (1990; see Supplementary Material 1 and Supplementary Figure 1). To ensure the pertinence of Winter's proportional model in the present study the measure of h (for each subject) was also obtained with a force platform (AMTI© OR6-7-2000; frequency of acquisition: 500 Hz/software BioAnalysis© see also Supplementary Material 2). This measurement was compared to that obtained from the video analysis using the anthropometric tables of Winter (1990) (see Supplementary Material 2).

### Basic characteristics of the VSJs

The most correct execution among 3 VSJs, for each subject, was chosen for analysis. The decision was made on the respect of the instructions given to execute the jump (initial stabilization of a semi-squatted position, arms akimbo during the jump, and initialization of the push-off without countermovement). This was systematically determined from the recorded videos of the jumps. As a further control, the measured *h*-values of the VSJ were compared to those obtained in other studies with skilled jumpers realizing maximal VSJs (e.g., Bobbert et al., 2008).

The heights of the jumps were measured as defined in Figure 1 and Supplementary Material 2 and the time length of the push-off phases as in Supplementary Material 3. To confirm the measurements the VSJ height from each subject was obtained with a force platform (see Section Data Acquisition).

**Figure 3:**
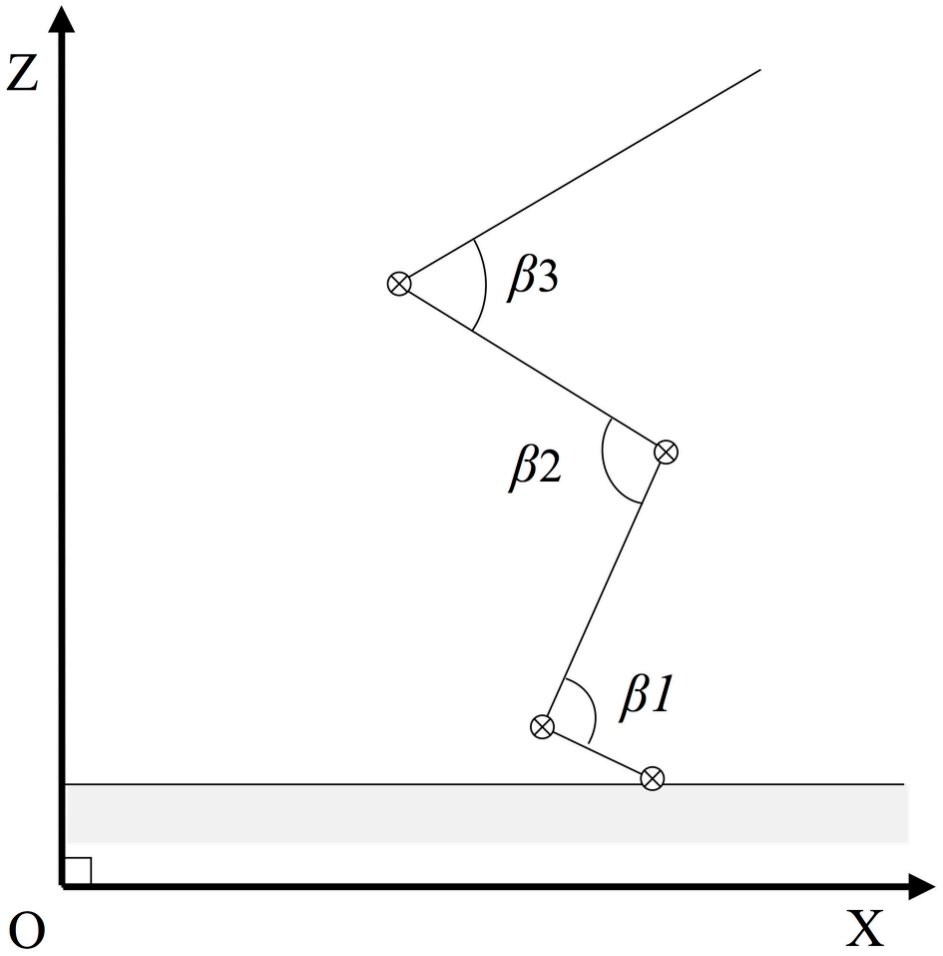
**Articular angles of the jumper**. The model of the jump (see Figure 2) includes three rotation axis (⊗) corresponding to three articulations (hip, knee, and ankle joints) thus to three articular angles βi.

The pattern of movement was also determined by measuring the opening of the articular angles (β) of the hip, the knee, and the ankle (Figure 3). To this end the variation (varia) of a given articular angle at each instant (t) of the push-off was taken in consideration [varia_t_ = (β_t+1_ − β_t−1_)∕2dt].

### Dynamics of coordination among segments

In a maximum VSJ, the rotation of the segments during push-off must lead to the translation of the jumper's MC to a maximal, vertical and linear velocity at take-off (starting from a static initial position; e.g., Van Ingen Schenau, 1989; see also Figure 2).

The movements of the segments were characterized at each instant of the push-off by: (a) the effect of the angular *velocity* of a segment i on the vertical velocity of the jumper's MC, this effect being quantified by: C_v_ = ${\overset{\cdot }{\theta }}_{\text{i}}\cdot {\cos\theta }_{\text{i}}$; (b) the effect of the angular *acceleration* of the segments on the vertical acceleration of MC, being quantified by: C_a_ = $\text{d}\left({\overset{\cdot }{\theta }}_{\text{i}}\cdot {\cos\theta }_{\text{i}}\right)∕\text{dt}$; and (c) the *efficiency* in the production of useful power (P_u_) at the jumper's MC by the rotation of the segments (Supplementary Material 1).

Concerning point (c) it should be kept in mind that the rotation of the segments may produce some “lost” power (P_l_), which does not contribute to the goal of the jump (see for example: Bobbert and van Soest, 2001). On this basis the produced muscular power (P_musc_) is the sum of P_u_ and P_l_. This leads to the expression of the energetic efficiency of the segments rotation as the ratio (R) between useful and muscular power, i.e., R = P_u_∕P_musc_ (see Supplementary Material 1).

#### Determination of possible segmental coordination types

To determine the presence of possible different coordination types the influence of the segmental movements on the vertical velocity and acceleration of MC was performed. It was found that an initial phase (T1) in the curve of the MC vertical velocity remained close to 0 m/s and was followed by a second phase (T2) in which the velocity of MC became definitely positive and increased through time. This confirmed previous observations (e.g., Bobbert and van Soest, 2001).

In the T2 phase possible early negative and later positive values of C_v_ (see Section Dynamics of Coordination among Segments) were searched in the curves of each segment, leading to the determination of consecutive different phases of C_v_ in all segments. Breaking points in the variations of the segmental C_a_ were searched in the curves determined by the following equation: $\left[{\text{d}\left({\overset{\cdot }{\theta }}_{\text{i}}\cdot {\cos\theta }_{\text{i}}\right)∕\text{dt}}_{\left(\text{t}+1\right)}-\;{\text{d}\left({\overset{\cdot }{\theta }}_{\text{i}}\cdot {\cos\theta }_{\text{i}}\right)∕\text{dt}}_{\left(\text{t}-1\right)}\right]∕2\text{dt}$.

The T1 and T2 phases in C_v_ and the breaking points in C_a_ for each segment led to a possible comparison among jumpers in order to suggest the hypothetical existence of a common dynamics of coordination and, consequently, to identify three sequential segment coordination types (to be called Ty_i_, Ty_inter_, and Ty_fin_) in each jump.

#### Coordination and energetic efficiency

The effects of the segmental rotation on the vertical velocity and acceleration of the jumper's MC led to the determination of consecutive types of coordination during push-off. The instantaneous energetic efficiency R (see Section Dynamics of Coordination among Segments and Supplementary Material 1) was calculated to search for a possible relation between R and the evolution of the coordination.

### Statistical analysis

Several statistical tools have been used according to the different aims of the study.

#### Basic characteristics of the jumps (pattern of movement)

The determination of the jumpers' pattern of movement (i.e., the opening of the articulations; see Section Basic Characteristics of the VSJs) was made following two steps. At first it was observed, for each articulation of a jumper, the instant at which a continuous increase above the average value of the angular variation was apparent. Then, for each segment, the test of homogeneity of Buishand (1982, 1984) was applied using the software Khronostat 1.01© to indicate a statistically significant breaking point in the curves of segmental angular velocity.

#### Dynamics of coordination among segments

The analysis of the two time periods that characterized the rise of MC during push-off (T1 and T2, see Section Determination of Possible Segmental Coordination Types) was determined by observing the instant at which the vertical velocity of the jumper's MC remained positive and increased. This was confirmed by the test of Buishand.

The presence of negative or positive C_v_ (in T2) was determined and the breaking points in C_a_ were analyzed by the test of Buishand (see Section Determination of Possible Segmental Coordination Types and Supplementary Material 1).

#### Coordination and energetic efficiency (R and a_MCz_)

The possible relation between the variation of the energetic efficiency of the segmental movements and the dynamics of the coordination of the segments during push-off was verified by the coefficient of correlation of Spearman between R and the vertical acceleration of the jumper's MC (a_MCz_; output of the segments rotation).

## Results

**Figure 4:**
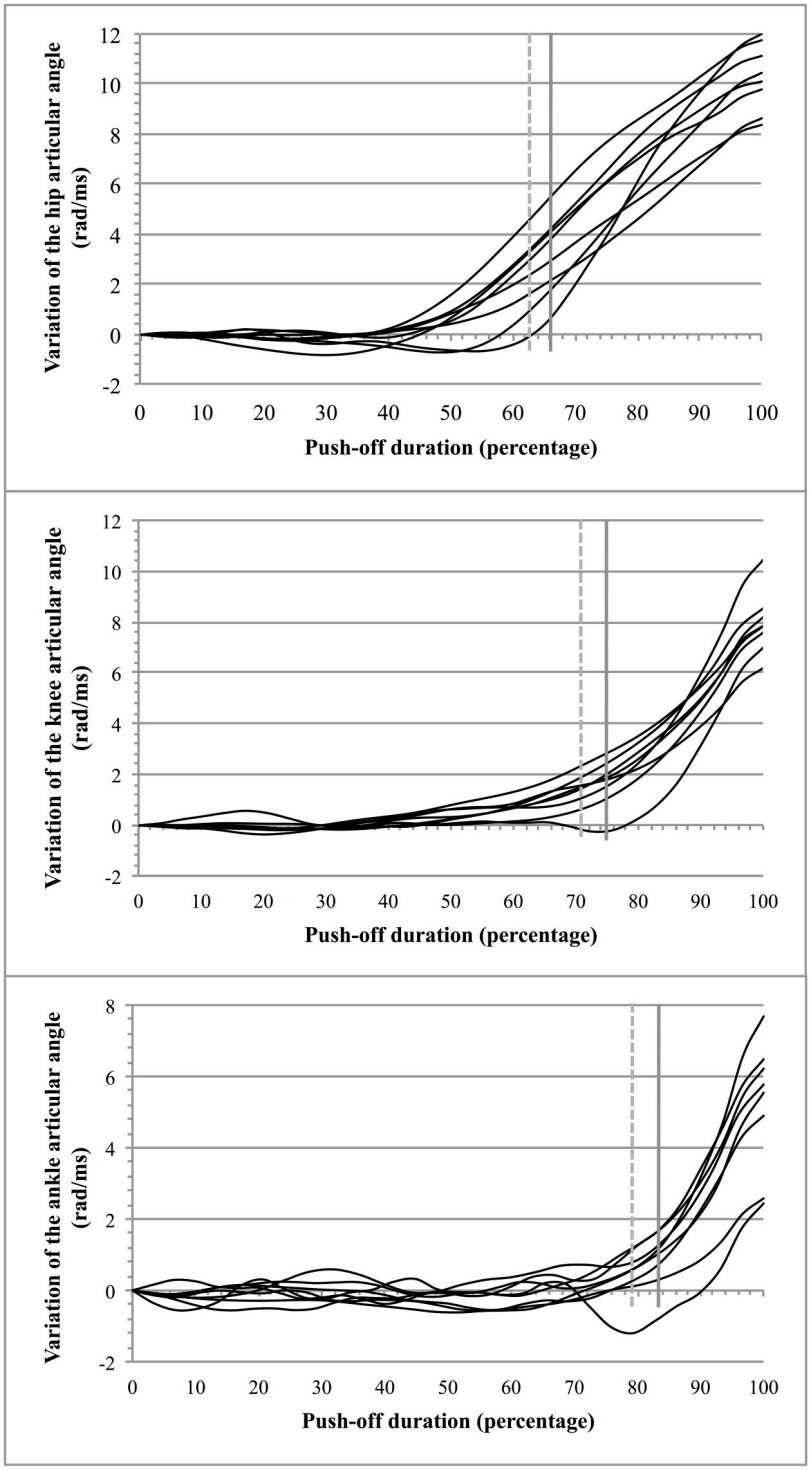
**Opening of the articular angles during push-off**. For a given articular angle βi (hip, knee, and ankle): the vertical dotted gray line indicates the average breaking point obtained from the test of Buishand and the vertical full gray line indicates the average instant from which the opening of a given articular angle increases beyond the mean value of variation of the corresponding articular angle.

The average jump height was 0.34 ± 0.06 m (*n* = 8) for an average push-off lasting 0.29 ± 0.02 s. Breaking points in the curves of the segmental articular angles were observed at a time instant that showed thereafter a continuous increase above the average value of the angular variation. The time instants at which these breaking points appeared (Figure 4)^1^ were found at: 66.3 ± 3% (hip), 75 ± 6% (knee), and 83.3 ± 5.1% (ankle) of the total time duration. A similar pattern of movement (Figure 4) was confirmed with the test of Buishand that showed quite comparable breaking points in the opening of the articulations (each breaking point of the curves was found to have a value of *p* < 0.001). The values were: 63.4 ± 3.3% for the hip, 71.7 ± 6% for the knee, and 79.2 ± 4.8% for the ankle. The two procedures (observation and test of Buishand) led to congruent results (the difference between the two procedures was of 3.4 ± 1.4%).

### Consecutive intersegment coordination

During push-off the rotation of the segments did not immediately provide a clear and continuous rise of the jumper's MC (the movements of the segments were not sufficient for such a rise and/or tending to neutralize each other at the beginning of the push-off). As it may be seen in Figure 4, two periods of push-off were observed that agreed with the data of Bobbert and van Soest (2001) (each breaking point of the curves was found to have a value of *p* < 0.001): (a) an initial period (T1 = 0.13 ± 0.02 s; i.e., 43% of the total push-off time) in which the vertical velocity of the jumper's MC remained close to 0 m/s and was not durably positive; and (b) a second period (T2 = 0.16 ± 0.03 s; i.e., 57% of the total push-off time) starting at the moment at which the velocity of MC became definitely positive. The effect of the segmental rotation on the vertical displacement of MC (both velocity and acceleration) led to the determination of three consecutive coordinations during T2.

**Figure 5:**
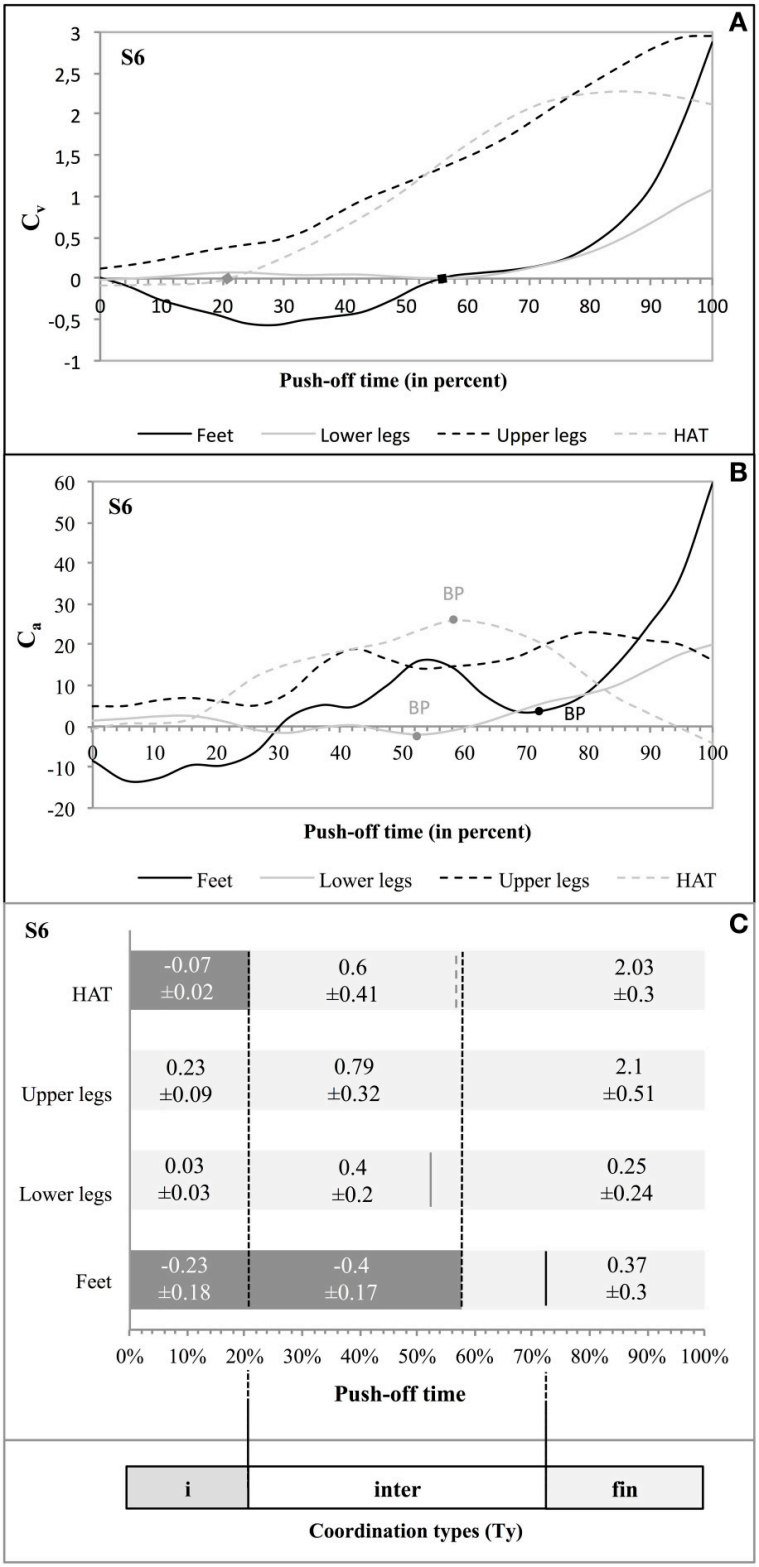
**Coordination types (Ty_i_, Ty_inter_, and Ty_fin_) during push-off in S6**. The curves C_v_ as function of time **(A)** show the influence of each segment of the jumper on the vertical velocity of his MC. The 2 squares on the axis of the abscissa indicate the passage from a negative C_v_ to a positive C_v_ of a given segment on the vertical velocity of MC. The curves C_a_ as function of time **(B)** show the influence of each segment of the jumper on the vertical acceleration of his MC; the breaking points are given when the *p*-value gives a significant probability (*p* < 0.05; see Supplementary Material 4). The bar chart **(C)** supplies the evolution of C_v_ as function of time for each segment. The mean values of C_v_ (± standard deviation) are also given. The doted vertical black lines (crossing the whole bars) indicate the phases of C_v_ for which these values are given. The vertical lines crossing a single bar indicate any breaking point (BP) identified by the Buishand test (full black line = BP feet; full gray line = BP lower legs; and dotted gray line = BP HAT). On this base, three consecutive types of coordination were determined, with: **(A)** Ty_i_ with 2 negative segmental C_v_ and no BP point in C_a_; **(B)** Ty_inter_ with an evolution in the segmental C_v_ from 1 negative influence to none and 3 BP in C_a_; and **(C)** Ty_fin_ during which each C_v_ was positive, and without any BP in C_a_.

Taking as an example jumper S6 (Figure 5) the influence of the segment rotation on the vertical velocity of the jumper's MC presented three distinct phases (Figure 5A). In the initial phase of C_v_ (from 0 to 21% of time) 2 segments (feet and HAT) did not influence positively the vertical velocity of MC. In the following phase of C_v_ (from 21 to 57.9% of time, i.e., 36.9% of time) 1 segment (feet) did not positively influence the vertical velocity of MC. In the final phase of C_v_ (from 57.9 to 100% of time; i.e., 42.1% of time) each segment of the jumper (feet, lower legs, upper legs, and HAT) showed a positive influence. The test of Buishand showed that the effects of the segmental rotation on the vertical acceleration of MC produced statistically significant breaking points at, respectively, 52.6% of time for the lower legs (*Q* = 8.73; *p* < 0.0001), at 57.9% for the HAT segment (*Q* = 8.95; *p* < 0.0001), and at 73.6% for the feet (*Q* = 6.63; *p* = 0.002) (Figure 5B). None of the breaking points observed in C_a_ appeared during the first phase of C_v_, 2 of them did so during the second phase of C_v_, and 1 during the last phase of C_v_ (Figure 5C). This led to the determination of 3 *coordination types* (Ty): (a) Ty_i_ with 2 negative segmental C_v_ and no breaking point in C_a_; (b) Ty_inter_ with the passage of 1 negative segmental C_v_ to 0 and 3 breaking points in C_a_; and (c) Ty_fin_ with each segmental C_v_ being positive and no breaking point in C_a_ (Figure 5C).

The dynamics of the movements found in S6 was similar in the other 7 subjects even if there was a definite variability in the realization of the three coordination types (Ty_i_, Ty_inter_, and Ty_fin_, see: Supplementary Figure 2). Thus: (a) Ty_i_ showed 1 or 2 negative C_v_ and no breaking point in C_a_; (b) Ty_inter_ was characterized by a decrease in the number of negative C_v_ and/or several breaking points in C_a_; (c) in Ty_fin_ each C_v_ was positive without any breaking point in C_a_ (see Supplementary Figure 2).

### Coordination and energetic efficiency

**Figure 6:**
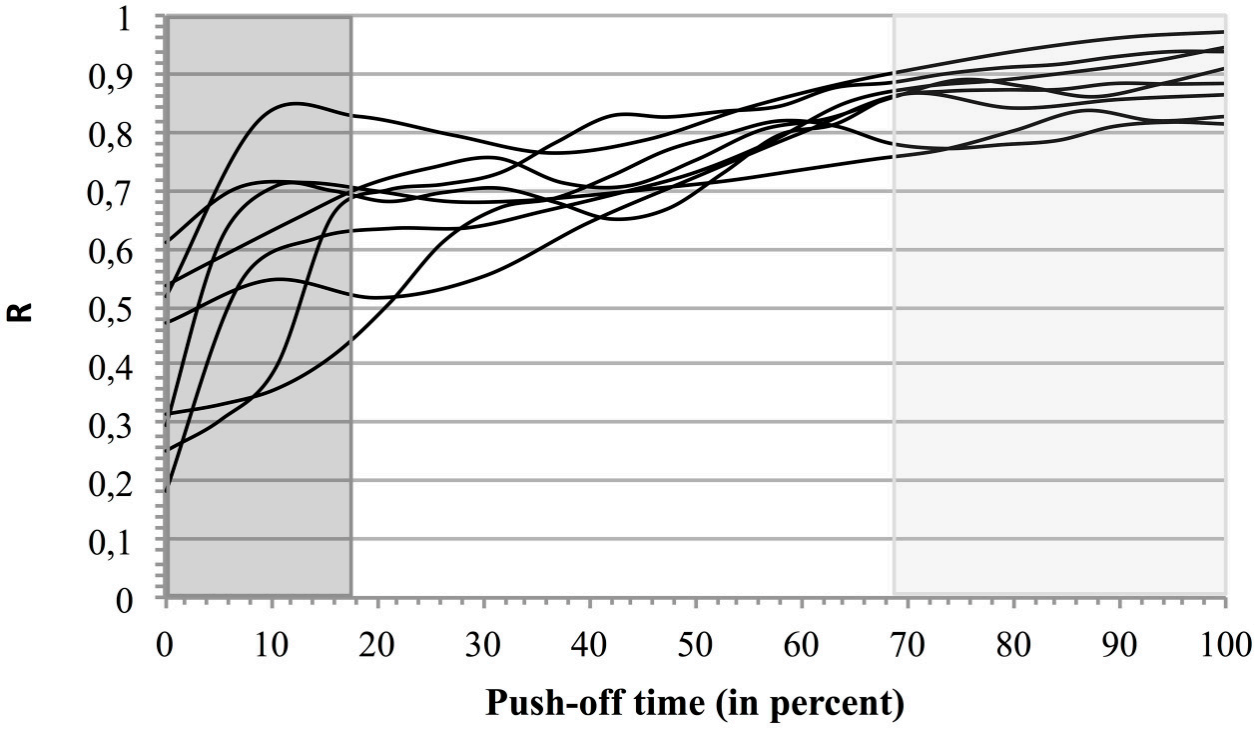
**Energetic efficiency R in 3 types of coordination**. For each jumper the curves of R as function of time are presented on a background indicating the mean identified segmental coordination types (percent of the push-off time). The first type (Ty_i_) is indicated by a dark gray background, the second (Ty_inter_), by a white background, and the third one (Ty_fin_), by a light gray background.

When the three types of coordination (Ty_i_, Ty_inter_, and Ty_fin_) were compared to the corresponding energy ratio R the changes of coordination type (indicated in dark gray, white, and light gray backgrounds; Figure 6) evolved in parallel with the improvement of R.

The coefficient of correlation of Spearman between R and the vertical acceleration of the jumper's MC (a_MCz_) was positive for each subject with a *p* ≤ 0.007 (Table 1).

**Table 1:** **Coefficient of correlation of Spearman between R and of a_MCz_ (m/s^2^)**.

	**S1**	**S2**	**S3**	**S4**	**S5**	**S6**	**S7**	**S8**
**Rho (R; a_MCz_)**	0.65	0.92	0.88	0.6	0.77	0.93	0.73	0.46
***p*****-value**	0.006	< 0.0001	< 0.0001	0.005	0.0004	< 0.0001	0.0009	0.007

The coefficient of correlation of Spearman (Rho) was calculated between R (the instantaneous energetic efficiency between useful and muscular power) and the vertical acceleration of the jumper's MC (a_MCz_). For each subject the coefficient was positive with a p-value of at least p ≤ 0.007.

**Figure 7:**
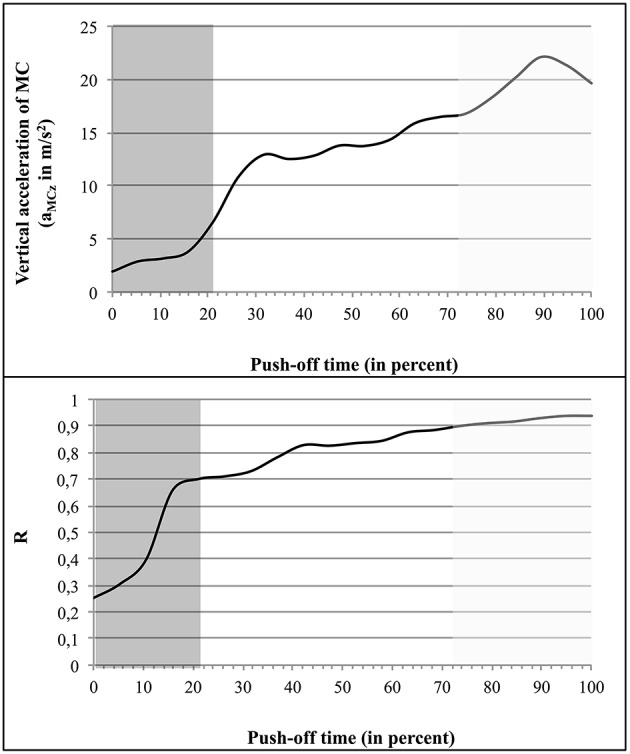
**Variation of a_CMz_ and R as function of time: example of the jumper S6**. The first coordination type (Ty_i_) is indicated by a dark gray background, the second (Ty_inter_), by a white background, and the third one (Ty_fin_), by a light gray background.

Figure 7 illustrates this correlation in the case of S6, showing both a_MCz_ and R as function of time (regression line: *y* = 0.0356x + 2.1518).

The mean values of both instantaneous R and vertical acceleration of the jumper's MC (a_MCz_), for each type of coordination, are given in Table 2.

**Table 2:** **Means values of R of a_MCz_ (m/s^2^) as function of the coordination type**.

**Jumps**	**Coordination type 1 (Ty_i_)**		**Coordination type 2 (Ty_inter_)**		**Coordination type 3 (Ty_fin_)**	
	**Mean R**	**Mean a_MCz_**	**Mean R**	**Mean a_MCz_**	**Mean R**	**Mean a_MCz_**
S1	0.70 ± 0.04	15.6 ± 2	0.78 ± 0.02	16.7 ± 0.9	0.83 ± 0.01	30.9 ± 8.1
S2	0.67 ± 0.22	14.50 ± 3.82	0.79 ± 0.03	21.70 ± 0.55	0.90 ± 0.07	25.14 ± 2.19
S3	0.49 ± 0.15	6.10 ± 2.97	0.78 ± 0.03	13.39 ± 0.51	0.79 ± 0.02	16.54 ± 1.53
S4	0.54 ± 0.21	7.89 ± 3.26	0.74 ± 0.07	13.55 ± 2.04	0.88 ± 0.005	20.73 ± 0.98
S5	0.52 ± 0.04	18.95 ± 2.5	0.77 ± 0.09	28.70 ± 1.05	0.86 ± 0.005	34.20 ± 2.72
S6	0.26 ± 0.1	3.66 ± 1.79	0.79 ± 0.07	14.38 ± 2.20	0.91 ± 0.01	20.81 ± 1.12
S7	0.59 ± 0.05	9.43 ± 2.92	0.72 ± 0.02	18.32 ± 2.10	0.85 ± 0.04	18.86 ± 1.24
S8	0.45 ± 0.23	12.83 ± 2.63	0.73 ± 0.09	15.12 ± 1.25	0.91 ± 0.02	17.07 ± 1.69

For each type of coordination (Ty_i_, Ty_inter_, and Ty_fin_) the table presents the mean values of both energetic efficiency between useful and muscular power (R), and vertical acceleration of the jumper's MC (a_MCz_).

In spite of the presence of some variability in R and a_MCz_ values, from one subject to another, the data systematically showed that the mean value of R increased from one type of coordination to the subsequent one (Table 2). The same trend was observed in the case of the instantaneous R and a_MCz_ (Table 1 and Figure 7). For example, in S6 the mean value of R (from 0 to 1) was of 0.26 ± 0.1, then of 0.79 ± 0.07, and finally 0.91 ± 0.01; the mean value of a_MCz_ (in m/s^2^) was initially 3.66 ± 1.79, then 14.38 ± 2.20, and finally 20.81 ± 1.12.

## Discussion

The present study concerns the dynamics of the coordination among four body segments during the push-off of a VSJ. The main hypothesis suggested the presence of a common dynamics among jumpers, even if differences in the segmental movements were probable.

### Basic characteristics of the VSJ (push-off time, height, and pattern of movement)

The mean value of the push-off time, lasting for 0.29 ± 0.02 s, and the mean height performance (0.34 ± 0.06 m) were in accordance with the average values already reported in the literature (e.g., Bobbert et al., 2008)^2^. The jumping skill of the subjects volunteering in the present study was confirmed by the opening of the articular angle of the hip earlier than that of the knee or the calf (see Section Results).

### Dynamics of inter-segmental coordination and motor control

The results showed the presence of *common* characteristics in the subjects' segmental movements during the push-off (see Section Consecutive intersegment coordination and Supplementary Figure 2, Supplementary Material 4). Thus, after the initial period of the push-off (T1), during which the segmental rotations provided a low vertical velocity of MC and not always with a positive value, it followed a second period (T2) during which the vertical velocity of the MC was increased and remained positive. During T2, and considering the four segments as a whole, three consecutive coordination types were observed (see Section Consecutive Intersegment Coordination and Supplementary Figure 2, Supplementary Material 4). This dynamics of coordination led to an increase in the number of segments influencing positively the vertical velocity of MC (from 2 to 3 in Ty_i_ and systematically 4 in Ty_fin_). The intermediate coordination type, Ty_inter_, participated to this evolution and showed breaking points in the segmental contributions to the vertical acceleration of MC (Supplementary Figure 2).

This *common dynamics of coordination* may imply the intervention of a programmed control of the movement (optimized through experience) by the impulses transmitted to muscles from the central nervous system. This may happen even if the observation of a given pattern of movement does not give sufficient information to deduce a corresponding pattern of stimulation by the central nervous system (e.g., Hudson, 1986; Robertson and Fleming, 1987; Bobbert and van Soest, 2001). It has been shown, in simulation studies (e.g., Prokopow et al., 2005), that the timing of muscles stimulation in VSJ has a determining influence on the quality of the push-off. In addition the very fast sequence of the three coordination types, shown in the present work (below 0.2 s), and above all the rapid changes in the segmental influences on the vertical displacement (velocity and acceleration) of MC during the second coordination type, Ty_inter_, may evoke the intervention of a mechanism of regulation during push-off. This support the idea of a distal modulation as it has been proposed by van Soest and Bobbert (1993) and Bobbert et al. (2013). This implies feedbacks from muscle to muscle, on the basis of an established cerebral and/or cerebellar motor program and of a relation strength-length-velocity. This might instantaneously regulate the jump by the viscoelastic properties of the muscle-tendon couple. Such modality of regulation presents the advantage to involve mechanisms of control much faster than that imputable to cerebellar circuitries in forward and inverse internal models (both kinematic and dynamic models) (e.g., Wolpert et al., 1998; Katsnelson, 2003). However, the slower motor velocity observed at the beginning of the push-off might also suggest a potential role of the neural feedback loops that would thus fade through the time of push-off.

**Figure 8:**
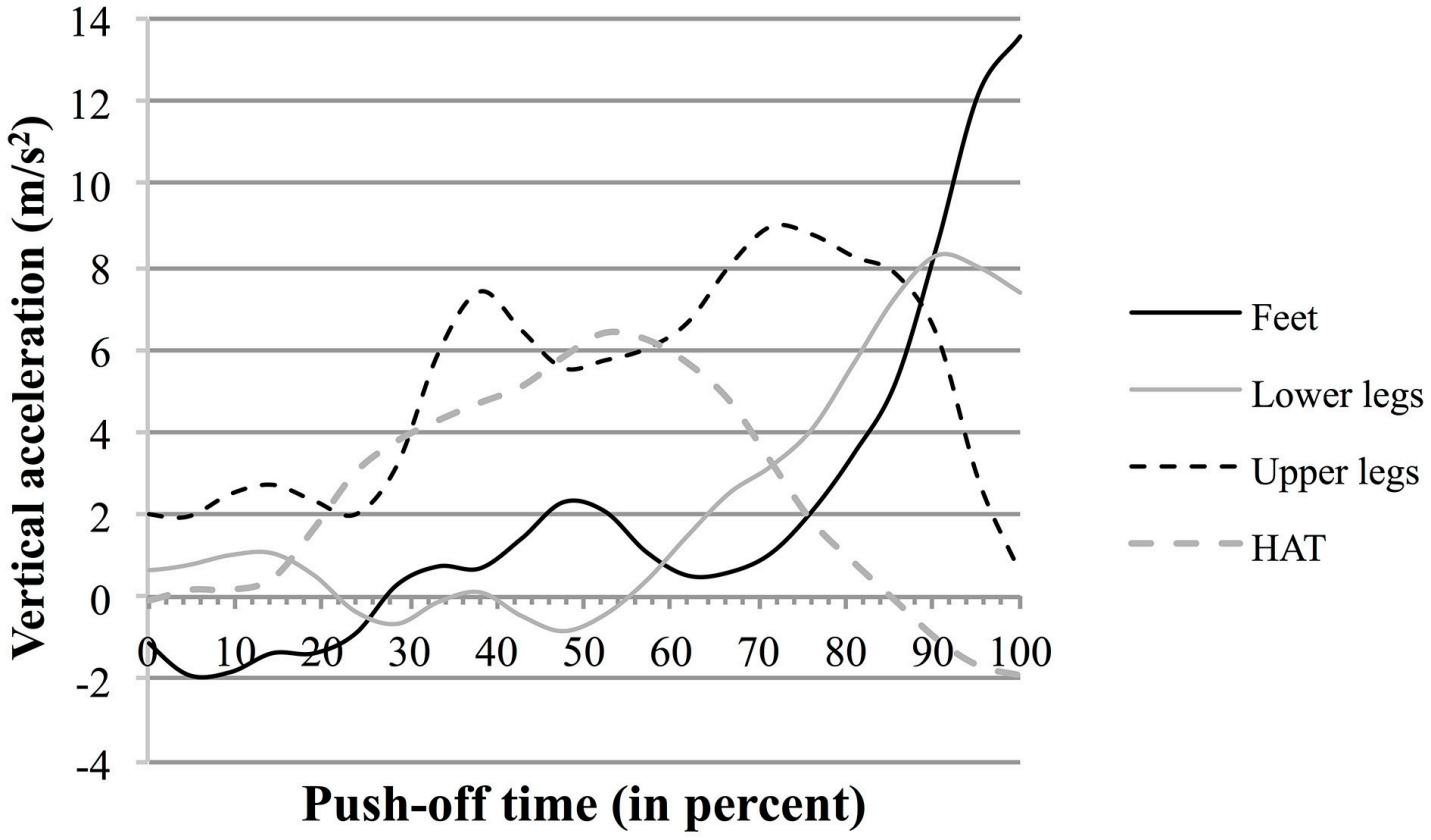
**Vertical acceleration of a jumper's segments as function of time during push-off : example of the jumper S6**. The full and dotted black lines, and the full and dotted gray lines represent the curves as function of time (percent of the push-off phase time length) of the vertical acceleration of the jumper's feet, upper legs, lower legs, and HAT, respectively.

The data shown in Supplementary Figure 2 and concerning the segmental participation to the vertical acceleration of the MC are compatible with such hypothesis. For example in S6 (see also Figure 5B) the breaking points show that the influences of the feet and of the lower legs evolve inversely to that of HAT. This is emphasized in Figure 8 showing the curves of the vertical component of the acceleration of the jumper's segments during push-off. Thus, the vertical component of the acceleration of the lower legs segment increased ca. 50% of push-off time, while it started to decrease in HAT. Similarly the vertical component of the acceleration of the feet increased to 70% of push-off time, while it started to decrease in the upper legs segment.

### Common motor control and inter-individual variability

The possible distal regulation of the push-off, following a given pattern of neural stimulation, is compatible with the observed inter-individual variability in segmental movements (see Supplementary Figure 2) provided that a *common referential element* for the regulation is present (see in the following).

Such variability may be due to a different cerebral programming of the VSJ or may be a function of the segment geometry and/or of the muscular capacities of the jumper. It may also be suggested that the initial posture may also play a role. Initial posture is established by the instructions given to the jumper by the experimenter (Figure 1). However, even if it would be possible to repeatedly set up a knee angle at exactly 90°, it should not be pertinent to do so, essentially because imposing a strict initial posture might influence the performance of the individual and his/her effort intensity (e.g., Sanders and Wilson, 1992). On the other hand keeping a given posture requires a dynamic process in which the subject's MC may oscillate randomly (e.g., Gurfinkel and Shik, 1973). As a consequence the jumpers begin their push-off starting from similar but not identical postures. In the case of a VSJ achieved from an initial free position, as in the present experiments, the posture variations observed in the videos were not very important. This suggests that the push-off might be regulated at the peripheral level only (from different initial positions of the segments and different muscular stretching), and consequently that a source of intra-individual variability might contribute to inter-individual variations in the segmental movements during push-off.

The regulation of the movement during push-off must be performed as function of a reference. Such a reference may be a “referential” movement determined from the initial stimulation of the muscles (van Soest and Bobbert, 1993). It should however be considered that from an initial posture a jumper must give to his/her MC the fastest vertical velocity at the instant of take-off. This must be done by producing the greatest useful power, at each instant of the push-off, thus by maintaining the MC vertical (theoretically without fluctuations) to the point of application of the ground reaction force. Such position of the MC may thus represent a value of reference to regulate the movement (Gurfinkel, 1973; Massion, 1992).

**Figure 9:**
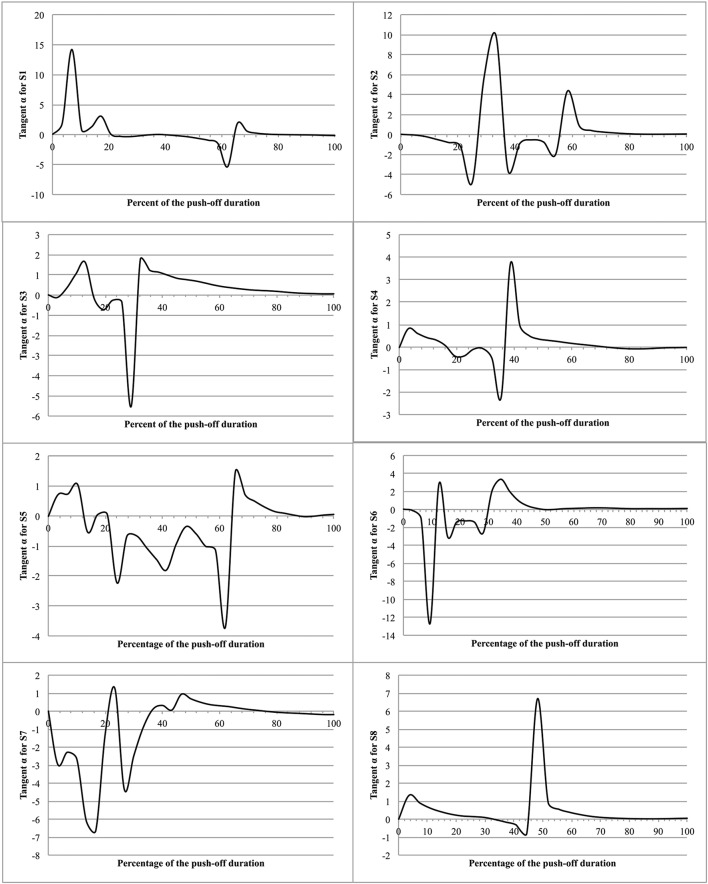
**Tangent α as function of time during push-off**. The figure presents the eight jumps as function of the percent of the push-off time. The ordinate is the tangent to the α angle made by the velocity vector of the MC (V_MC_) with the vertical passing by the point of application of the ground reaction force (tangent α = V_MCx_/V_MCz_).

To examine the possible fluctuations in the position of the MC, relative to such vertical, the results of the present experiments allow to determine at each instant of the push-off the angle α made by the velocity vector of the MC ($\vec{{\text{V}}_{\text{MC}}}$) with the vertical passing by the point of application of the ground reaction force, knowing that: tang(α) = V_MCx_∕V_MCz_. It is interesting to note that any divergence of the velocity vector to the vertical reference produces an adjustment that reduces the divergence, thus suggesting *a persistent regulation of equilibrium* during the jump (Figure 9).

Moreover, the evolution of the segment coordination during T2 suggests a *continuous increase of energetic efficiency* (Tables 1, 2), i.e., an increasing utilization of produced muscular power as useful power. This finding and the evolution of tang(α) indicate that jumpers generate an increased vertical velocity of MC while reducing the power losses by maintaining their MC vertical to the point of application of the ground reaction force. The conclusion is that the evolution of the segments rotation, from an initial posture, is function of a less energetic cost.

The ensemble of the present and published data suggests the following conclusions: (a) the push-off control is dependent upon a motor cerebral stimulation adapted to the jump and to the jumper's segments characteristics; (b) the continuous regulations of the jump may be probably related to the jumper's perception of the position of MC relative to the ground reaction force; (c) such regulations are made to reduce energetic losses; (d) the control of the initial posture is determinant in the dynamics of the segment coordination. It should be emphasized that the ensemble of the results make possible the regulation of the VSJ in a very short time, even below 10 ms^3^, thus in physiologically extreme conditions of control.

## Author contributions

The authorship criteria (author guidelines) were met by PF, RM, TR, AG, and EF. Conceived and designed the experiments: PF, RM, TR, AG, and EF. Performed the experiments: PF, RM, and EF. Analyzed the data: PF, RM, TR, AG, and EF. Contributed reagents/materials/analysis tools: PF, RM, TR, AG, and EF. Wrote the paper: PF, RM, and EF.

### Conflict of interest statement

The authors declare that the research was conducted in the absence of any commercial or financial relationships that could be construed as a potential conflict of interest.

## Footnotes

^1^Presented as percent of the time period of the push-off phase to normalize differences among subjects.

^2^The mean height performance was comparable to the literature even if such comparisons are quite difficult to make because of the different techniques used to measure jump height (Aragón-Vargas, 2000). Thus, Bobbert et al. (2008) reported that gymnasts could jump up to 0.41 ± 0.05 m, while Bobbert et al. (2006) reported a height of 0.284 m (no SD was given) in adults having performed in volleyball or gymnastics. It should be noted that in the latter studies the height of jump was defined as the difference between the height of the jumper's MC at the peak of the jump and that of the subject standing up before push-off; understandably the measure of the jump height gave higher values than that measured in the present study (Figure 1). These observations finally support the idea that the subjects of the present study did their best, thus producing a maximal effort.

^3^This can be deduced for example from subject S6 (Supplementary Figure 2) where the BP of the lower legs segment is at 52.6% of time duration and the BP of HAT is at 57.9% (Section Consecutive intersegment coordination), i.e., a difference of 5.3% (total time duration of 0.19 s). In real time the regulation between two segments happened in ~0.009 s.
