# Supplementary material for: Fast Regulation of Vertical Squat Jump during Push-Off in Skilled Jumpers
Source: Front Physiol. 2016 Jul 19;7:289. doi: 10.3389/fphys.2016.00289 (PMC4950838; doi:10.3389/fphys.2016.00289)
Supplement: Supplementary file 1 [file Presentation1.pdf]

Supplementary Material

Fast Regulation of Vertical Squat Jump during push-off in skilled jumpers

Fargier P[1]*, Massarelli R[1], Rabahi T[1,2], Gemignani A[3], Fargier E[1]

[1]University of Lyon, University Claude Bernard Lyon 1, Inter-University Laboratory on Human Movement Biology (LIBM, EA 7424), Centre for Interdisciplinary Research in Sport (FED 4272), 69622 Villeurbanne cedex, France

[2] Laboratoire de Conception, Optimisation et Modélisation des systèmes (LCOM, EA 7306), équipe Emotion-Action, Université de Lorraine, Ile du Saulcy, 57006 Metz Cedex 1

[3]Dipartimento di Patologia Chirurgica, Medica, Molecolare et dell'Area Critica, Università degli Studi, Pisa, Italy

*** Correspondence:** Fargier P, University Claude Bernard Lyon 1, Inter-University Laboratory on Human Movement Biology (LIBM, EA 7424), Centre for Interdisciplinary Research in Sport (FED 4272), 69622 Villeurbanne cedex, France.

patrick.fargier@univ-lyon1.fr

1 Segmental dynamics of coordination

During a VSJ push-off (Figure 1), an anaerobic transformation of internal potential energy in muscular energy (E_{musc}) occurs in the recruited muscles, thus producing muscular power ($P_{musc} = dE_{musc}/dt$). As a consequence the segments rotate and in response to the reaction to the ground the combination of these segmental rotations leads to the displacement of the jumper's mass center (MC) and thus to the transmission of power to the MC (P_{MC}). Where: $P_{MC} = m \cdot \overrightarrow{a_{MC}} \cdot \overrightarrow{v_{MC}} = m \cdot (d\overrightarrow{v_{MC}}/dt) \cdot \overrightarrow{v_{MC}}$; m being the mass of the jumper, $\overrightarrow{a_{MC}}$ is the acceleration of MC, and $\overrightarrow{v_{MC}}$ its velocity. Part of this power useful to obtain the goal of the jump (P_u) allows the vertical displacement of the MC: $P_u = m \cdot a_{MCz} \cdot v_{MCz}$; a_{MCz} and v_{MCz} being respectively the vertical components of the acceleration and of the velocity vectors of the MC. Thus: $v_{MCz} = P_u/(m \cdot a_{MCz})$. At take-off the kinetic energy of the jumper's MC is: ($E_k = {}^1/_2 \cdot m \cdot v_{MCz}^2$) and, after take-off, this is transformed in potential energy ($E_{pot} = m \cdot g \cdot h$). The peak of the flight phase of the jumper's MC (h_{max}) can thus be deduced considering the principle of the conservation of energy and disregarding the frictional forces of air (negligible) on the jumper. Thus: $h_{max} = v^2_{MCz_{tTO}}/2g$; where t_{TO} is the instant of take-off, and g is the value of the acceleration of gravity. Consequently, $h_{max} = P^2_{u_{tTO}}/(m \cdot a_{MCz_{tTO}})^2 \cdot 2g$. Finally, the height of jump depends on the useful power transmitted to the jumper's MC by the segmental rotations.

The object of the study was to define the influence of the segmental rotations upon the vertical velocity of the MC at each instant of the push-off during a maximal VSJ. This implies to clearly

distinguish the relation between the movements of each segment and that of the jumper's MC. The resulting physical system and the corresponding model of the jumper (Figure 2) lead to the calculation of the velocity of the jumper's MC from its momentum ($\vec{p}$) with $\vec{p} = m \cdot \overrightarrow{v_{MC}}$ where m is the mass of the jumper and $\overrightarrow{v_{MC}}$, the velocity vector of his MC. This shows the link between the movements of the segments and that of the jumper's MC. With regards to the vertical velocity, the momentum of the jumper's MC is thus the sum of the momenta of the segments with $p_{MCz} = m \cdot v_{MCz} = \sum m_i \cdot v_{MCzi}$ where p_{MCz} and v_{MCz} are respectively the vertical component of the momentum and of the velocity of the jumper's MC, m_i is the mass of the segment i, and v_{MCzi} the vertical component of the velocity vector of the mass center of the segment i.

Each v_{MCzi} results from the rotational velocity of the segment i and from the velocity imposed by the segment below. Thus: $v_{MCzi} = v_{iz} + V_{(i-1)z}$; where v_{iz} is the vertical velocity of the MC of the segment i and $V_{(i-1)z}$ the vertical velocity that the segment below the segment i imposes to segment i. The calculations are operated from the segmental angular velocity of each segment ($\dot{\theta}_i$). The values of anthropometric parameters are also required, i.e.: (a) the distance between each mass center of a segment and the corresponding (distal) rotation axis (l_i) and (b) the length of the segment (L_i). These parameters are shown in supplementary Figure 1.

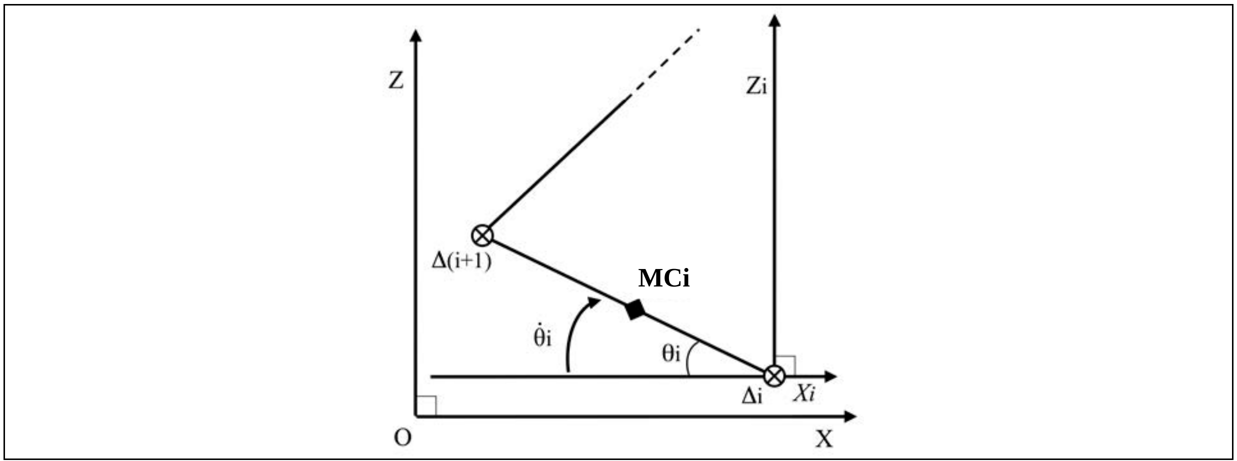

Supplementary Figure 1. Landmarks for the calculation of the linear velocity of a MCi. According to the model shown in Figure 2**,** MCi is the mass center of a given segment i, Δ_i is the rotation axis of the segment i, and $\Delta_{(i+1)}$ that of the segment above. On this basis l_i is the distance [Δ_i, MCi] and L_i is the length of the segment i (i.e.: the distance [Δ_i, $\Delta_{(i+1)}$]). These landmarks allow to specify the linear velocity of the MCi, the linear velocity of Δi, and the linear velocity of $\Delta_{(i+1)}$, respectively $\vec{vi}$, $\vec{Vi}$, and $\overrightarrow{V(i+1)}$. It must be noted that as long as the jumper is in interaction with the ground, then $\vec{Vi} = \vec{0}$.

Thus:

$$v_{MCz1} = l_1 \cdot \dot{\theta}_1 \cdot \cos\theta_1$$

$$v_{MCz2} = (l_2 \cdot \dot{\theta}_2 \cdot \cos\theta_2) + (L_1 \cdot \dot{\theta}_1 \cdot \cos\theta_1)$$

$$v_{MCz3} = (l_3 \cdot \dot{\theta}_3 \cdot \cos\theta_3) + (L_2 \cdot \dot{\theta}_2 \cdot \cos\theta_2) + (L_1 \cdot \dot{\theta}_1 \cdot \cos\theta_1)$$

$$v_{MCz4} = (l_4 \cdot \dot{\theta}_4 \cdot \cos\theta_4) + (L_3 \cdot \dot{\theta}_3 \cdot \cos\theta_3) + (L_2 \cdot \dot{\theta}_2 \cdot \cos\theta_2) + (L_1 \cdot \dot{\theta}_1 \cdot \cos\theta_1)$$

Considering that $p_{MCz} = m \cdot v_{MCz} = \sum m_i \cdot v_{MCzi}$ it is thus possible to write that:

$$v_{MCz} = k_1(\dot{\theta}_1 \cdot \cos\theta_1) + k_2(\dot{\theta}_2 \cdot \cos\theta_2) + k_3(\dot{\theta}_3 \cdot \cos\theta_3) + k_4(\dot{\theta}_4 \cdot \cos\theta_4) \text{ (Equation 1)}$$

In Equation 1 the only variable parameters are the segmental angular velocities and the respective cosine of the corresponding segmental angles. The coefficients k_i depend on the anthropometric characteristics of the jumper, with l_i and L_i as defined in supplementary Figure 1, and m_i being the mass of a given segment i. The anthropometric values (li and Li) were determined from Winter (1990). Then:

$k_1 = [(m_1 \cdot l_1) + (m_2 \cdot L_1) + (m_3 \cdot L_1) + (m_4 \cdot L_1)]/\sum m_i$
$k_2 = [(m_2 \cdot l_2) + (m_3 \cdot L_2) + (m_4 \cdot L_2)]/\sum m_i$
$k_3 = [(m_3 \cdot l_3) + (m_4 \cdot L_3)]/\sum m_i$
$k_4 = (m_4 \cdot l_4)/\sum m_i$

Thus, Equation 1 shows that the product of the segmental angular velocity ($\dot{\theta}_i$) by the cosine of the corresponding angle (θ_i) is the *first determining parameter* to define the contributions of each segmental angular rotation to the vertical velocity of the jumper's MC at each instant of the push-off. Consequently the *second determining parameter* is the derivation as function of time of $\dot{\theta}_i \cdot \cos\theta_i$, i.e.: $d(\dot{\theta}_i \cdot \cos\theta_i)/dt$. From Equation 1, and knowing that a_{MCz} is the vertical acceleration of the jumper's MC, it is thus possible to write:

$$a_{MCz} = k_1 \frac{d(\dot{\theta}_1 \cdot \cos\theta_1)}{dt} + k_2 \frac{d(\dot{\theta}_2 \cdot \cos\theta_2)}{dt} + k_3 \frac{d(\dot{\theta}_3 \cdot \cos\theta_3)}{dt} + k_4 \frac{d(\dot{\theta}_4 \cdot \cos\theta_4)}{dt} \text{ (Equation 2)}$$

The evolution, at each instant of the push-off, of the values of each $\dot{\theta}_i \cdot \cos\theta_i$ and $d(\dot{\theta}_i \cdot \cos\theta_i)/dt$ shows the influence of each segmental rotation on the vertical velocity of the jumper's MC, thus on the useful power (P_u) transmitted to the jumper's CM.

The rotation of the segments may however also produce some power not contributing to the goal of the jump as the rotation of a segment produces, by definition, a horizontal component of velocity whose combination with the other segmental movements may participate to a horizontal displacement of the jumper's MC. Such power (P_X) at the MC level would then be useless to reach the goal of the jump (where: $P_X = a_{MCx} \cdot v_{MCx}$ and a_{MCx} and v_{MCx}, respectively, represent the horizontal component of the acceleration and of the velocity vectors of the jumper's MC; see also Figure 2). The relative velocity of the MC of a given segment (MCi) to the velocity of the jumper's MC may also influence the produced power not contributing to the goal of the jump ($P_{MCi/MC}$). More precisely: $P_{MCi/MC} = d(Ec_{MCi/MC})_t/dt$; where $Ec_{MCi/MC}$ is the relative kinetic energy of the mass center of a given segment with respect to that of the jumper's MC. The muscular power, at a given instant of the push-off, is thus the sum of the useful power and of two types of « lost » power (considering the goal of the jump). This leads to the calculation of the energetic efficiency of the segmental rotations at a given instant of the push-off with the ratio of the produced useful power by the produced muscular power: ($R = P_u/P_{musc}$). This ratio is thus the *third determining parameter* to be taken into consideration.

Equations 1 and 2 may thus be used for calculating the useful power transmitted to the jumper's MC, as $P_u = m \cdot a_{MCz} \cdot v_{MCz}$. Similar equations allow the calculation of the power corresponding to the

horizontal displacement of the jumper's MC, i.e. $P_X = a_{MCx} \cdot v_{MCx}$. The terms of this product are thus obtained, respectively from Equations 2 and 1, by changing each $\cos\theta_i$ in $\sin\theta_i$. Equation 1 also gives the basis for calculating the power due to the relative velocity of the mass center of a given segment with respect to the velocity of the jumper's MC, i.e.: $P_{MCi/MC}$. Knowing that $P_{musc} = P_u + P_X + P_{MCi/MC}$, and considering the goal of the jump, the calculations of P_u, P_X, and $P_{MCi/MC}$ may thus determine the energetic efficiency of the segmental rotations at a given instant of the push-off, i.e.: $R = P_u/P_{musc}$.

2 Height of a VSJ

The height of the VSJs has been calculated from the data allowed by the video analysis. From Appendix 1 the height of a VSJ may be defined as follows:

$$h = (p_{MCz}/m)^2 \cdot (1/2g) \text{ (Equation 3)}$$

In Equation 3, p_{MCz} is the value of the vertical component of the momentum of the jumper's MC, m is the weight of the jumper, and g is the value of the acceleration of gravity. Considering that $p_{MCz} = m \cdot v_{MCz} = \sum m_i \cdot v_{MCzi}$ it is possible to demonstrate (see Equation 1) that the calculation of V_{MCz} from the videos of the jumps requires the use of anthropometric tables to determine: (a) the distance between each mass center of a segment and the corresponding (distal) rotation axis (l_i), (b) the length of the segment (L_i), and (c) the mass of the segment (m_i).

These values were determined, on the base of the height and the mass of the jumper, from the anthropometric tables of Winter (1990). Le mass center of HAT was calculated step by step taking in consideration: (a) the definition of the MC of a set of several elements: (b) the geometry (symmetries, lengths) of HAT and of its components; (c) the respective positions of the mass centers of these components.

The use of the anthropometric tables was important in the calculations, as the present study focuses on the influence of the segmental rotations on the vertical displacement of the jumper's MC (see previous § 1). It was thus necessary to verify the pertinence of the anthropometric tables (Winter, 1990), and to this end the heights of the VSJs were also calculated from data given by a force platform (AMTI© OR6-7-2000; frequency of acquisition: 500 Hz/software BioAnalysis©). Being A (in N.s), the "area of the impulse" given by the platform, the height of jump can be written as follows:

$$h = (v_{MCz}^2/2g) = (A/m)^2/2g \text{ (Equation 4)}$$

In Equation 4, $A = \int_{t0}^{t_{TO}} (GR_Z - P).dt$; where t0 is the instant at which the push-off begin, t_{TO} is the instant of take-off, GR_z is the value of the vertical ground reaction force recorded by the force platform, and P, the jumper's weight. From the data provided by the force platform, A was calculated by numerical integration, which led to the calculation of h.

The resulting heights obtained with the two methods described above were compared and were found to be similar (the mean difference was 3 ± 2 %), thus showing the pertinence of the use of Winter's anthropometric tables (see supplementary Table 1).

Supplementary table 1. Vertical velocity of the jumpers' MC at take-off ($V_{MCz_{(tTO)}}$) and heights (h) of the VSJs.

		S1	S2	S3	S4	S5	S6	S7	S8
**VW**	$V_{MCz_{(tTO)}}$ **(m/s)**	2.89	2.53	2.22	2.8	2.73	2.53	2.77	2.16
	**h (m)**	0.43	0.33	0.25	0.4	0.38	0.32	0.39	0.24
**FP**	$V_{MCz_{(tTO)}}$ **(m/s)**	2.9	2.58	2.26	2.83	2.8	2.58	2.78	2.22
	**h (m)**	0.43	0.34	0.26	0.41	0.4	0.33	0.39	0.25

Comparison of the measurement of $V_{MCz_{(tTO)}}$ and h obtained from the video analysis using the anthropometric tables of Winter (1990) (called "video Winter", VW) to that obtained with a force platform (FP).

3 Time duration of push-off phase

The push-off phase of a VSJ starts at t0, when the segments of the jumper start rotating, and ends at take-off. The instant of take-off was determined by the modifications of the system dynamics produced by the suppression of the interaction between feet and the ground support. This was done by calculating the angular acceleration of a given segment at a given instant t ($\ddot{\theta}_{i(t)}$), with $\ddot{\theta}_{i(t)} = \left(\dot{\theta}_{i(t+dt)} - \dot{\theta}_{i(t-dt)}\right)/2dt$. The suppression at take-off of the reaction force exerted by the support induces a strong variation of $\ddot{\theta}_{it}$; the variation may then be determined.

Alternatively the take-off instant may be calculated considering α = (V_{MC}, OZ) (see Figure 2) obtained from the tangent given by the ratio $(\sum \text{mi Vix})/(\sum \text{mi Viz})$, and supposing that the linear velocity of the rotation axis on the feet at the distal extremity of the feet (v_1) is nil (in contact with the ground, see Figure 2). The calculation of α is valid only for the push-off phase. At take-off, the vertical component of $\overrightarrow{v_1}$ undergoes a strong variation. Thus the calculation of alpha shows a strong increase in the variation rate of α after the instant of take-off.

The comparison of the results obtained from the two methods (from $\ddot{\theta}_{it}$ and from α) thus allows the determination of the instant of take-off.

4 Intersegmental coordination

After the initial phase T1 (T1 = 0.13 ± 0.02 s; i.e.: 43% of the total push-off time), in which the velocity of the jumper's MC remained close to 0 m/s, it followed a second phase in which the velocity became clearly positive and increased (T2 = 0.16 ± 0.03 s; i.e.: 57% of the total push-off time). The effect of the segment rotation on both the vertical velocity (C_v) and the vertical acceleration (C_a) of the jumper's MC was examined in T2. The examination of C_v and C_a lead to the determination of three coordination types (Ty) and of an inter-individual variability in their realization (supplementary Figure 2).

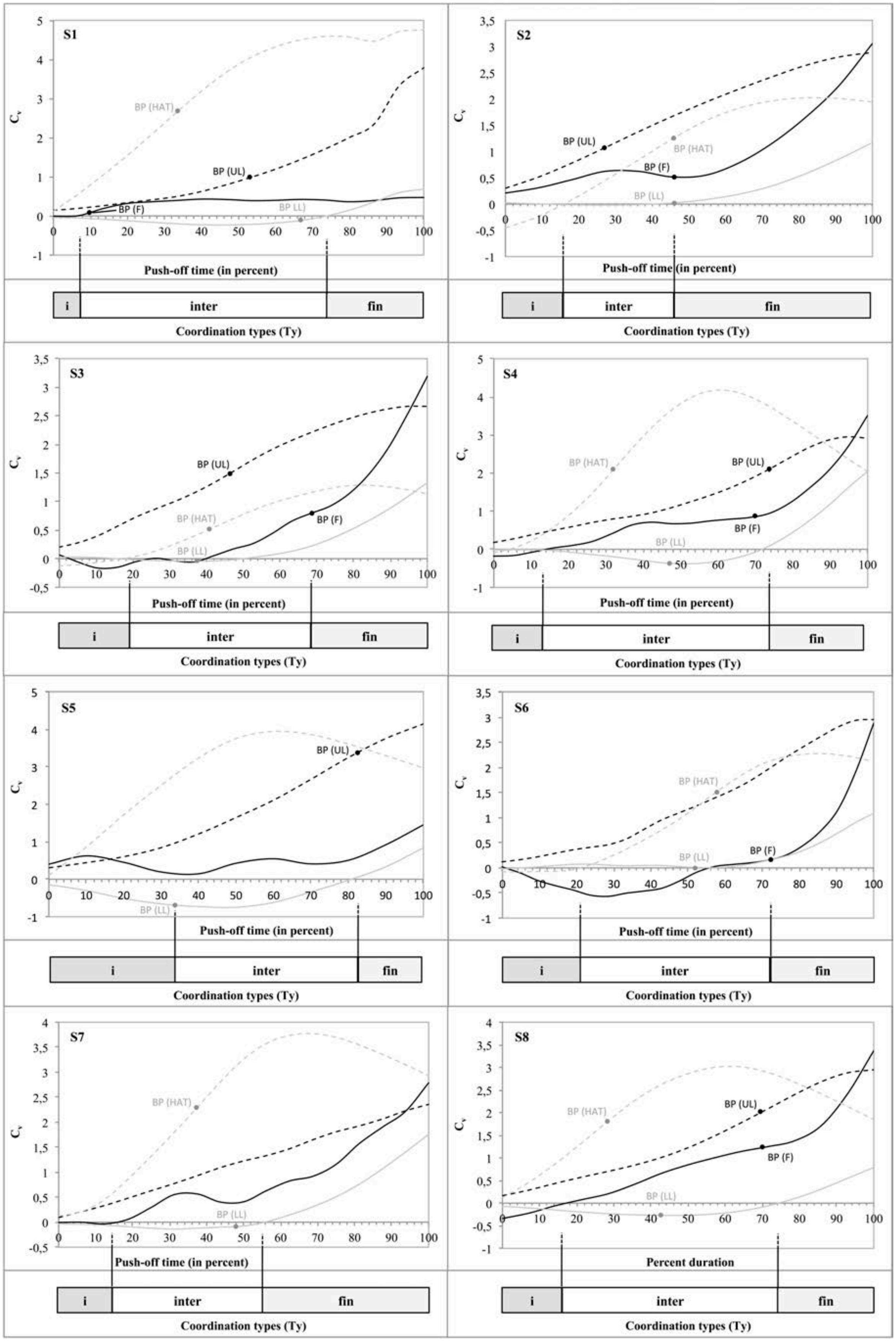
S1
BP (HAT)
BP (UL)
BP (F)
BP (LL)
Push-off time (in percent)
i
inter
fin
Coordination types (Ty)
S2
BP (UL)
BP (HAT)
BP (F)
BP (LL)
Push-off time (in percent)
i
inter
fin
Coordination types (Ty)
S3
BP (UL)
BP (HAT)
BP (F)
BP (LL)
Push-off time (in percent)
i
inter
fin
Coordination types (Ty)
S4
BP (HAT)
BP (UL)
BP (F)
BP (LL)
Push-off time (in percent)
i
inter
fin
Coordination types (Ty)
S5
BP (UL)
BP (LL)
Push-off time (in percent)
i
inter
fin
Coordination types (Ty)
S6
BP (HAT)
BP (LL)
BP (F)
Push-off time (in percent)
i
inter
fin
Coordination types (Ty)
S7
BP (HAT)
BP (LL)
Push-off time (in percent)
i
inter
fin
Coordination types (Ty)
S8
BP (HAT)
BP (UL)
BP (F)
BP (LL)
Percent duration
i
inter
fin
Coordination types (Ty)

Supplementary Figure 2. Consecutive types of segmental coordination during the push-off of a SVJ. The curves C_v as function of time (percent duration of T2, i.e.: the phase of the push-off during which the velocity of the jumper's MC was continuously positive and increased) show the influence of the rotation of each segment of a jumper (S1-8) to the vertical velocity of his MC with: feet = black full line; lower legs = grey full line; upper legs = dotted black line; and HAT = dotted grey line. The points on the curves (BP, with: feet = F; lower legs = LL; upper legs = UL; and HAT = head-arms-trunk) indicate each breaking point (when given by the Buishand with a p-value showing a significant probability of at least $p < 0.05$) in the influence of the segmental rotations on the acceleration of the jumper's MC. The vertical black line under the axis of the abscissa indicate the limits of the coordination types during push-off: Ty_i; Ty_{inter}; and Ty_{fin}.

The influence of the segmental rotation on the vertical velocity of the jumper's MC (C_v) showed systematically different phases (from 2 to 3 phases) regarding the negative and positive C_v (supplementary Figure 2). From one phase to the next one the number of negative C_v decreased and, during the last phase, each segmental C_v was positive. For example, in S5 (supplementary Figure 2), 2 phases were identified with: (a) 3 positive C_v (feet, upper legs, and HAT) and 1 negative C_v (lower legs), and (b) 4 positive C_v (each segment). Another example of the noticed general diminution of the negative C_v through time may be given in the case of S8 (supplementary Figure 2) with: (a) 2 positive C_v (upper legs and HAT) and 2 negative C_v (feet and lower legs); (b) only 1 negative C_v (lower legs); and (c) each segmental C_v being positive. Despite such inter-individual variations a dynamic was observed with an increase of the concomitant positive segmental C_v through time (T2).

The test of Buishand showed that the effects of the segment rotation on the vertical acceleration of the jumper's MC (C_a) presented in each jump at least 2 (from 2 to 4; see supplementary Figure 2) breaking points (BP) with p-values showing a significant probability ($p < 0.05$). For example, 2 BP were found in S7, in the C_a of HAT and in that of the lower legs (supplementary Figure 2), while 4 BP were found in S8 (thus 1 BP per segment). The results of the Buishand's test are given in supplementary Table 2.

Supplementary table 2. Results of the Buishand test regarding the segmental C_a.

Buishand test		S1	S2	S3	S4	S5	S6	S7	S8
F	Q	4.979	5.49	5.6	7.75		6.63		5.99
	p	0.002	< 0.001	0.04	< 0.001		0.02		0.02
LL	Q	5.994	4.59	8.21	7.51	4.12	8.73	6.5	4.78
	p	0.001	0.015	< 0.001	0.002	0.02	< 0.001	0.004	0.03
UL	Q	6.573	4.95	8.2	7.62	4.53			6.03
	p	< 0.001	0.002	< 0.001	< 0.001	0.008			< 0.001
HAT	Q	6.573	5.11	9.06	7.62		8.95	8.73	5.44
	p	< 0.001	0.001	< 0.001	< 0.001		< 0.001	< 0.001	0.01

The table presents the results obtained from the homogeneity test of Buishand (Q values) that identifies breaking points in the curves (e.g., Supplementary Figure 2), which describe the influence of the segment rotation on the vertical acceleration of the jumpers' MC. The Q values represent a probability of at least $p < 0.05$. The jumpers' segments are indicated by: F = feet; LL = lower legs; UL = upper legs; and HAT = head-arms-trunk.

These observations regarding C_v and C_a showed common trends among subjects beyond their inter-individual variability. They led to the identification of three coordination types in each jump (Ty; see supplementary Figure 2), limited by the passage of a segmental C_v from a negative value to a positive one and/or by a BP in a segmental C_a: (a) Ty_i showed 1 or 2 negative C_v and no BP; (b) Ty_{inter} was characterized by a decrease in the number of negative C_v and/or several breaking points in C_a; (c) in Ty_{fin}, each C_v was positive and there was no BP in the C_a.

5 References

Winter, D.A. (1990). *Biomechanics and motor control of human movement*. New York: Wiley & sons
